# Fibroin nanofibers production by electrospinning method

**DOI:** 10.3906/kim-2011-36

**Published:** 2021-08-27

**Authors:** Derya SALTIK ÇİRKİN, Metin YÜKSEK

**Affiliations:** 1 Institute of Pure and Applied Sciences, Faculty of Technology, Textile Engineering, Marmara University, İstanbul Turkey; 2 Faculty of Technology, Textile Engineering, Marmara University, İstanbul Turkey

**Keywords:** Electrospinning method, silk fibroin, nano material

## Abstract

Silk fibroin, which has many characteristic properties such as low inflammation reaction, biodegradation, suppleness, good antithrombogenic details, biocompatibility and high tensile strength is a very good candidate for biomedical applications. Electrospinning procures high surface area, porous, nanofiber dimension fiber generation, which is a plain method. An experimental study was carried out to produce nanofiber structure from silk fibroin by electrospinning and the electrospinning parameters for the spinning of uniform, continuous and silk fibroin fibers were optimized. As a result, the effect of variables of concentration, distance and applied voltage on the strength, thickness, surface structure, fiber diameter of nanomaterial was investigated. Then, in vitro cell viability of the silk fibroin mat was analyzed. It was seen that the strength, mat thickness, and fiber diameter increased with solution concentration rise. It was found that the values of the fiber diameter and tensile strength decreased with increasing distance. It was determined that the effect of distance varies depending on the concentration in the mat thicknesses. The tensile strength was affected inversely proportional the applied voltage rises and distance. It was found that the fiber diameter values decreased together with increasing applied voltage. At cell viability of silk fibroin mat was occurred high cell viability after 24 h, but it was obtained low cell viability at the 48th h.

## 1. Introduction

Silk is one of the oldest and most useful animal fibers known by people [1,2]. Silk is an animal fiber produced by certain insects that create a net around themselves and form their cocoons. The silk fibers are mainly composed of two proteins structure as fibroin in inner layer and sericin in outer layer [1]. Fibroin is the protein that forms the filaments of silkworm silk and provides unique physical and chemical properties to silk fiber. Sericins are the group of gummy proteins that bind the fibroin filaments [3]. The chemical compound of silk includes usually 72%–83% fibroin, 17%–28% sericin, 0.8%–1.0% oil and wax, 1.0%–1.4% dyestuff and ash and 11% water [1,4,5]. The amino acid structure of silk fibroin mainly consists of two nonpolar amino acids glycine (43%) and alanine (30%) [6,7]. It then consists of 12% serine and low amounts of cystine, acidic and essential amino acids [3,6]. The amino acid structure of the silk series is the opposite [7]. Primarily, polar amino acids serine (30%), aspartic acid, and the nonpolar amino acids glycine and alanine are present together at only about 22% [3,7]. Silk has a molecular weight (200–350 kDa or more) with bulky repeating modular hydrophobic domains blocked by small hydrophilic groups [2]. Silk fibers have a smooth surface. It is seen as a fibroin triangular structure with corners covered with sericin in its cross-section [8]. The fineness of the raw silk coming out of the silkworm as two filaments is 1.8–3 denier, and the fineness of a single silk filament with the sericin removed is 1–1.5 denier [4,9]. Silk filaments are the longest of natural fibers. The filament is between 1000 and 3000 m in a cocoon [4]. The filament can be drawn between 500 and 1200 m from the cocoon [9]. The diameter of raw silk fibers is between 12 µm and 30 µm. The fibers drawn from the outside of the cocoon are much thicker inside fibers. These are the ones secreted later, and they are thinner and uniform [4].

Natural silk fibers are dissolved only in a limited number of solvents, compared to globular proteins, because of the presence in fibroin of a great amount of intramolecular and intermolecular hydrogen bonds and its high crystallinity and specific physicochemical properties as wetting angle of 69° ± 3° [3]. Fibroin can be dissolved in concentrated aqueous solutions of acids (phosphoric, formic, sulphuric and hydrochloric) and high ionic strength aqueous salt solutions such as calcium chloride (CaCl_2_), zinc chloride (ZnCl_2_), lithium bromide (LiBr) and magnesium chloride (MgCl_2_) [10].

Silk fibroin (SF) is a natural biopolymer that has biocompatible, biodegradable, low inflammation reaction, flexibility, good antithrombogenic and spectacular mechanical properties [11,12]. SF porous materials have been widely investigated in controlled drug delivery system, anticoagulant blood materials, biosensors, artificial ligaments, artificial tendon, artificial skin, nerve repair and grafting, cornea repair and wound dress, etc. [13].

Many studies have been reported that the SF scaffolds provide excellent support for cell proliferation and can also use for various tissue engineering, including bone, cartilage, and blood vessel [14].

The world of nanomaterials includes a wide variety of interesting materials with exceptional physical and chemical properties and qualifications. These materials include zero-dimensional nanoparticles or quantum dots, one-dimensional nanowires, nanorods, nanofibers, and nanotubes, and two-dimensional nanosheets. Considered as nanomaterials with perfect latent applications, nanofibers distinguish oneself among the rest of the nanomaterials. One of the flashiest features of nanofibers is their extraordinarily high surface-area-to-volume ratio and high porosity, making them a solid and appealing candidate for many forward applications. The true proof of the importance of nanofibers can be witnessed from the many building blocks from which they can be synthesized to the range of applications in which they have been established to have a remarkable effect. Up to today, nanofibers have been prepared from an admixture of materials, such as natural polymers, synthetic polymers, carbon-based nanomaterials, semiconducting nanomaterials, and composite nanomaterials. Along with the quick develop in the synthesis and characterization of nanofibers over the past few years, tremendous efforts have been focused on exploring the potential functional applications of nanofibers, water, and environmental treatment, including energy generation and storage, biomedical engineering, and healthcare [15].

The electrospinning method is described as the method of forming the material by making smaller material under electrical and hydrodynamics forces [16]. The late 1500s, first example was William Gilbert’s magnetics and electrostatics event [17]. In 1745 Bose, spraying with glass capillary tip [18,19], J. F. Cooley, the first definition of electrospinning in 1902 [17], in 1934 Anton Formhals received the first patent for fiber production [20]. In 1978, Sim obtained fibers and these fibers were used in the air filter [20]. In 1986, Hayati, effects of environmental factor, in 1996, Reneker demonstrated that many polymeric types of nanofibers could be obtained [21]. In 2003, Fridrikh developed a mathematical equation to predict the diameter fineness of fibers [22, 23]. 

Synthetic materials that can support the proliferation, differentiation, and improvement of living mammalian cells into the desired tissue in vitro are of significant interest. Such materials, scaffolds, must supply structural promote and coactions that direct cell growth. Favourably, the scaffold material would be biocompatible, fully biodegradable, or absorbable, porous with high surface area, similar in mechanical properties to the target tissue, and sterilizable. Nanofibers are acknowledged particularly promising for tissue scaffolds because their size scale mimics the extracellular matrix [24].

Due to the biocompatible and mechanical properties of SF, it has been used by the fact that many investigators either alone or as a multicomponent mixture. At the beginning of these production methods, there is an electrospinning method [25–38]. The researchers used 12%–28% concentration and 2–4 kV applied voltage in their studies. At the end of produced, the researchers reached the fiber diameter quality of 12–1500 nm. As understood from the literature review, there have been many studies on making a silk fibroin cell viability test. According to the cell proliferation tests performed on silk fibroin structures, they found that cell proliferation was achieved up to 3–7 days and 3 weeks (endothelial cell silk fibroin). In this way, silk fibroin is a biopolymer suitable for development in terms of wound healing, blood vessels, bionanotextiles, and tissue engineering [39–42].

As a result of this study, the effects of concentration, distance, and applied stress parameters on tensile strength, mat thickness, mat surface, and fiber diameter changes were investigated. A cell proliferation test has been performed on the produced silk fibroin nanofiber structure. The highest strength, mat thickness, mat surface, and fiber diameter of silk fibroin nanofiber structures have been evaluated, and the effect of silk fibroin on cell proliferation has been observed.

## 2. Experimental

### 2.1. Material selection

Cocoons of *Bombyx mori* silkworm were kindly from Kozabirlik (Bursa, Turkey). Sodium carbonate (Na_2_CO_3_), ethanol (CH_3_CH_2_OH), formic acid (HCOOH, 98%) and calcium chloride unhydrate (CaCl_2_) were purchased from Merck (Darmstadt, Germany). Cellulose dialysis membranes (molecular weight cut off, MCWO: 12,000–14,000) were purchased from Sigma-Aldrich (St. Louis, MO, USA).

### 2.2. Method

#### 2.2.1. Dissolution process of silk fibroin

Silk cocoons were opened by cutting and cleared inside. Then the cocoons were cut pieces. The cocoons were degummed with 0.02 M Na_2_CO_3_ solution at 100 °C for 40 min and then washed three times with destilled water. Fibroin fibers were dried at 80 °C for 6 h and put in the desiccator for moisture absorption. Fibroin fibers were waited during 24 h. Fibroin fibers were dissolved in a ternary solvent system of CaCl_2_/H_2_O/CH_3_CH_2_OH solution (1:8:2 in mol ratio) for 1 h at 78 °C.

#### 2.2.2. Dialysis and centrifuge process

The fibroin solution was dialyzed against distilled water for 3 days by exchanging dialysis water at every 12 h to remove CaCl_2_/H_2_O/CH_3_CH_2_OH solution. The solution was centrifuged at 9000 rpm, 5 °C for 20 min. 

#### 2.2.3. Preparation of silk fibroin solid mass

The fibroin solution was put the beaker and stirred at 45–55 °C. After the water was completely removed, remaining solid mass was fibroin. The solid mass fibroin was waited 3 h in the incubator. 

#### 2.2.4. Preparation of the spinning solution

Fibroin was dissolved in formic acid (98%). The concentrations of solutions were 10–12.5–15% wt. The viscosity of the polymer solutions was determined by using viscometer (Brookfield DV-E Viscometer, Brookfield, WI, USA). The viscosity measurement was performed with S21 spindle at 100 rpm. The conductivity of the polymer solutions was measured by conductivity meter (WTW Cond 3110, Germany). All experimental study was carried out at room temperature. The viscosity and conductivity values of polymer solution are shown in Table.

**Table:** The viscosity and conductivity values of polymer solution.

Concentration of solution(% wt)	Viscosity value(cP)	Conductivity value(μS/cm)
10	46 +¯ 2	1447 +¯ 15
12.5	130 +¯ 27	1575 +¯ 20
15	239 +¯ 40	1764 +¯ 30

#### 2.2.5. Electrospinning process

A 10-mL plastic syringe was filled with fibroin solution for electrospinning. The electrospinning device (NE300 Nanospinner, Inovenso, İstanbul, Turkey) was used to produce nanofibers. Cylindrical collector surface was covered with grease-proof paper. The set parameters of electrospinning process such as applied voltage, distance between cylindrical rotating collector and the nozzle, feeding rate of the polymer solution were 35, 37, 40 kV; 15, 20, 25 cm and 0.3 mL/h, respectively. The velocity of cylindrical rotating collector was adjusted at 500 rpm. The experiments were performed at 26–28 °C.

#### 2.2.6. Morphology of nanofiber structure

The morphology of the electrospinning nanofibers was analysed with scanning electron microscope (SEM) images (FEI Sirion XL-30 SEM and Inspect S50 SEM, FEI/Thermo Fisher Scientific Inc., Waltham, MA, USA). Samples of 10 × 10 mm were taken for measurement. The samples were covered with gold. ImageJ software for measuring fiber diameters of images was used.

#### 2.2.7. Fouirer transform infrared spectroscopy (FTIR)

In the FTIR (Fourier transform infrared spectra) examination, the silk fibroin mats were obtained on Perkin Elmer-120 (Waltham, MA, USA). Samples of 10 × 10 mm were taken for measurement.

#### 2.2.8. Mechanical test

All nanofiber structures were cut to 50 × 10 mm (length × width) for the mechanical test. Instron 4411 universal test (Instron, Norwood, MA, USA) device was used to examine the mechanical properties of nanofiber structures. The piston speed was set 3 mm/min.

The thickness of nanofiber structures was measured with a Mitutoyo Digital Thickness Comparator (Mitutoyo, Kawasaki, Japan). Thickness measurements were made at 30 different points in both vertical and horizontal directions. 

#### 2.2.9. In vitro cell viability analysis

The experiments were used normal human/healthy human keratinocyte skin cell line (ATCC CRL-2404). The nutrient media was used 10% fetal bovine serum (FBS) and formula of ATCC containing 1% penicillin-streptomycin Dulbecco’s modified essential medium-F12 (DMEM F12). The cells were incubated in humidified incubator at 37 °C. The incubator had 5% carbondioxyde (CO_2_). The essences of nanofiber mats were prepared ISO 10993-12 standard, according to the described protocol. Samples were prepared as 0.1 g sample per 1 mL^–1^ nutrient media. Nanofiber mats were extracted by stirring in DMEM nutrient media at 24 h. The extracts were incubated in 100% concentration culture media, which were 37 °C, 5% CO_2_. The samples were observed at 24, 48 and 72 h. Tetrazolium [MTT- (3-(4,5-dimethylthiazol-2-yl)-2,5-diphenyltetrazolium bromide)] was used to determine cell viability. The absorbances were measured with Boyn DR200B microplate reader at 590 nm (Boyn Industrial, Hangzhou, China). The materials of tested were expressed as cell viability percentage compared to the negative control.

## 3. Results

### 3.1. Mechanical test

The silk fibroin nanofiber mats obtained were done mechanical test at 6 repetitive. The graphics were obtained by average. Concentration, distance and applied voltage different impacts of mats were compared at the graphics obtained. 

**Figure 1:**
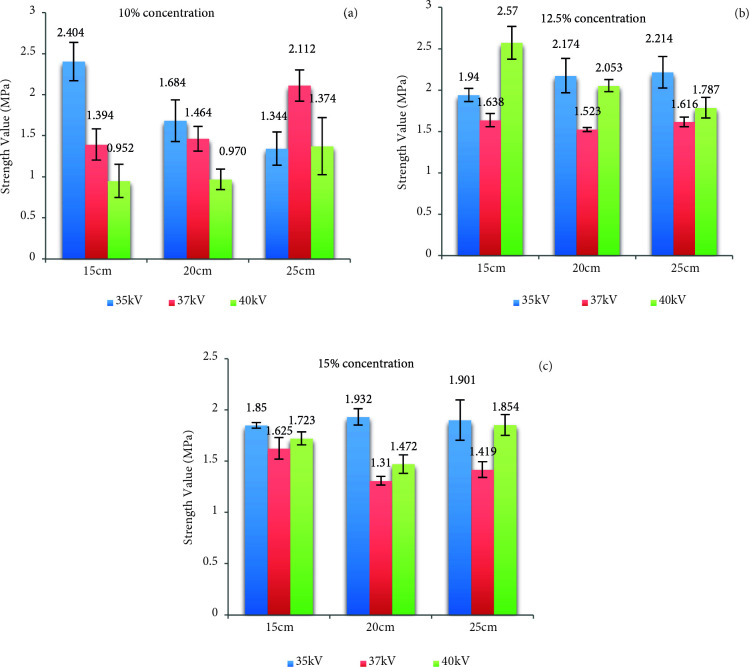
Strength value results of silk fibroin nanofiber mats, 10% (a), 12.5% (b) and 15% (c) concentration.

Silk fibroin nanofiber mats were produced three different values of concentration, distance and applied voltage. Strength value results of mats were indicated at Figure 1. 

At 10% concentration (Figure 1a), the highest tensile strength value was found at 35 kV applied voltage. The highest strength value among mats was observed at 15 cm distance and 35 kV applied voltage.

At 12.5% concentration (Figure 1b), the highest tensile strength value was one another close at 35 kV and 40 kV applied voltage. However, it has been seen the highest tensile strength value at 15 cm distance and 40 kV applied voltage. 

At 15% concentration (Figure 1c), the tensile strength value of mats had resulted one another close. At 35kV applied voltage was displayed the most superior strength value at all the distances. The highest tensile strength value mat was obtained at 35 kV applied voltage and 20 cm distance.

When all the results were examined (Figure 1), it was found that the concentration of 12.5% was the appropriate concentration to obtain the most strength mat. The tensile strength affected by applied voltage increase was inverse proportion to distance. When the literature was reviewed, it has been revealed that it should be evaluated by considering the differences in concentration, applied voltage, feeding rate, distance, test sample sizes, electrospinning device. It was observed that the strengths of silk fibroin nanofiber structures reached values between 10.3–12.5–18.9 MPa [43–45]. As mentioned before, the one-to-one comparison is not possible due to the high number of active parameters.

### 3.2. Thickness results of mats 

**Figure 2:**
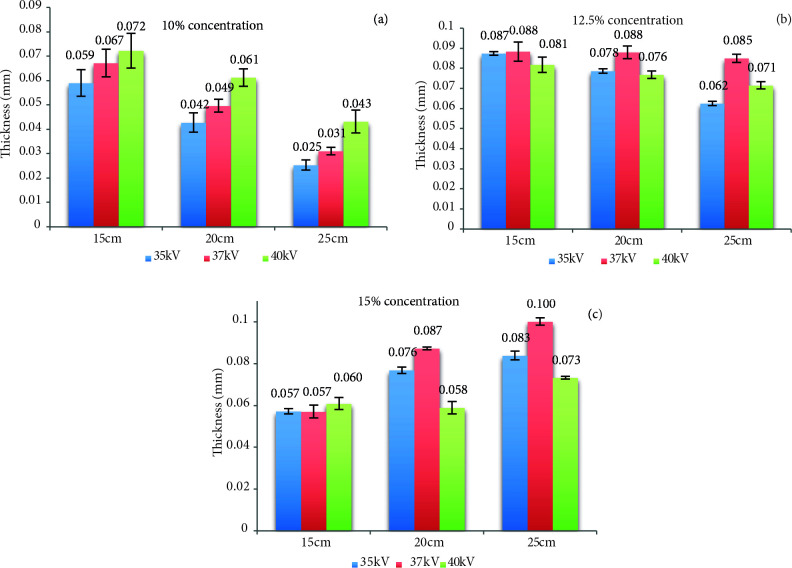
Thickness value results of silk fibroin nanofiber mats, 10% (a), 12.5% (b) and 15% (c) concentration.

Nanofiber mats were produced three different values of concentration, distance and applied voltage. The thickness value results of mats were indicated in Figure 2.

When the literature was examined, a comparison with the thickness values of the silk fibroin nanofiber structure has not been found. The mat thickness is necessary information for the tensile strength test results. Since the tensile strength is shown as MPa in the literature, thickness of mat can be calculated here. It is provided as additional information in our research. Therefore, a comparison has been made to the obtained silk fibroin nanofiber mats. In Figure 2, when thickness test results of all mats were investigated, the thickest mats were obtained at 12.5% concentration. At 10% concentration, the obtained mats had usually the lowest thickness value. 

### 3.3. FTIR analysis

**Figure 3:**
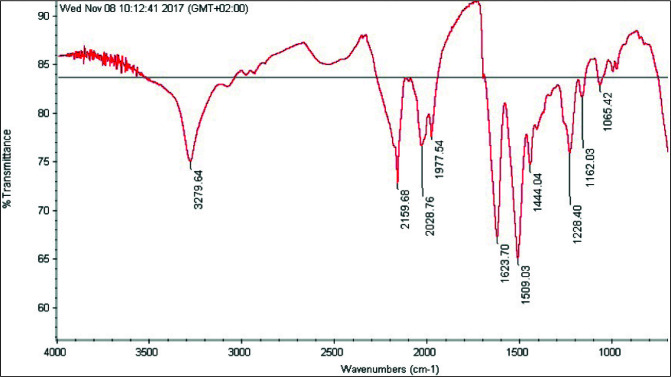
FTIR transmittance spectra of degummed silk fibroin.

**Figure 4:**
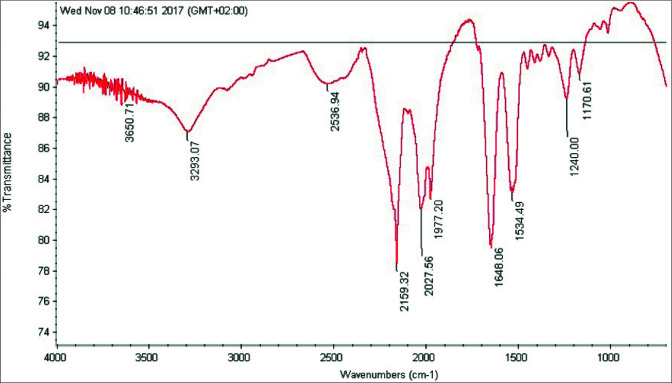
FTIR transmittance spectra of electrospinning produced silk fibroin nanofibers mat.

FTIR spectrums of degummed silk fibroin and electrospinning produced silk fibroin nanofibers mat were indicated in Figures 3 and 4.

Due to the existence of amide groups in silk protein, the self-givenness pulse strips around 1620 cm^–1^ were allocated to the absorption peak of the peptide backbone of amide I (C=O stretching), the strips around 1230 and 1444 cm^–1^ to amide III (C-N stretching), strips around 1513 cm^–1^ to amide II (N-H bending) and 694 cm^–1^ to amide IV. Overall, these self-givenness absorbance peaks prognosticate the creature of a hydrogen-bonded NH group. The molecular structure of silk fibroin is characterized by β-sheet absorption peaks around 1630, 1530, and 1240 cm^–1^, superficial coil quality absorption peaks at 1650 or 1645, 1550, and 1230 cm^–1^, and an α-helix absorption peak around 1655 cm^–1^. The intensity of peaks around 3300 cm^–1^ undulates in reply to hydrogen bonds [46].

According to the FTIR results (Figures 3 and 4), degummed silk fibroin was characterized by β-sheet absorption peaks 1623 cm^–1^ (amid I), 1509 cm^–1^ (amid II), 1228–1444 cm^–1 ^(amid III). Electrospinning produced silk fibroin nanofibers mat was characterized by random coil conformation absorption peaks at 1650 or 1645, 1550 and 1230 cm^–1^, and an α-helix absorption peak around 1655 cm^–1^. In the literature review, approximate and same value peaks were found with FTIR spectra. It has been proved in FTIR results that the obtained nanofiber structures consist only of silk fibroin [46–47].

These band shifts occurred because of the distinct hydrogen bonding states produced by the different conformations adopted by protein chains [47]. 

### 3.4. SEM results

**Figure 5:**
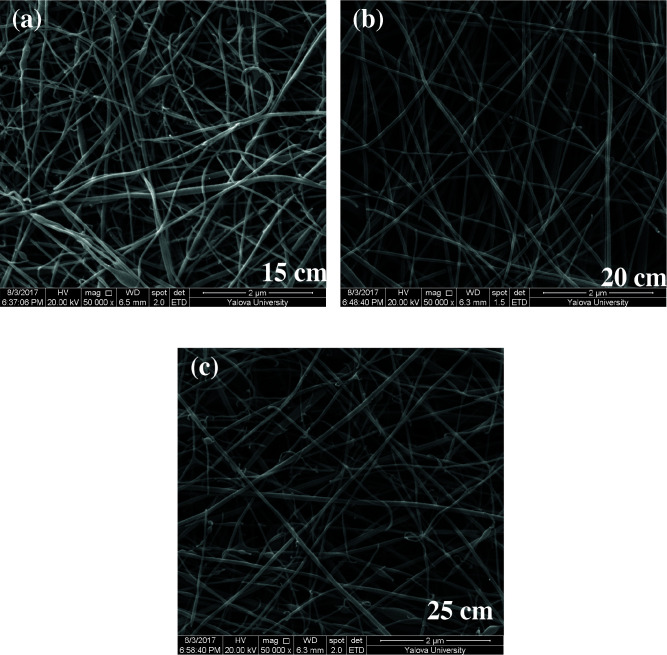
SEM images of silk fibroin nanofiber mats with 10% by weight and applied voltage 40 kV [15 cm (a), 20 cm (b) and 25 cm (c), respectively] ( 50000).

**Figure 6:**
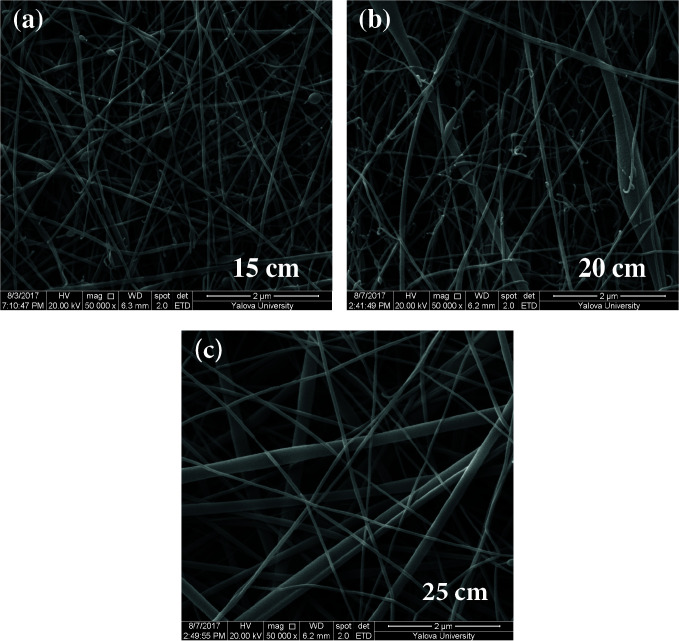
SEM images of silk fibroin nanofiber mats with 10% by weight and applied voltage 37 kV [15 cm (a), 20 cm (b) and 25 cm (c), respectively] ( 50000).

**Figure 7:**
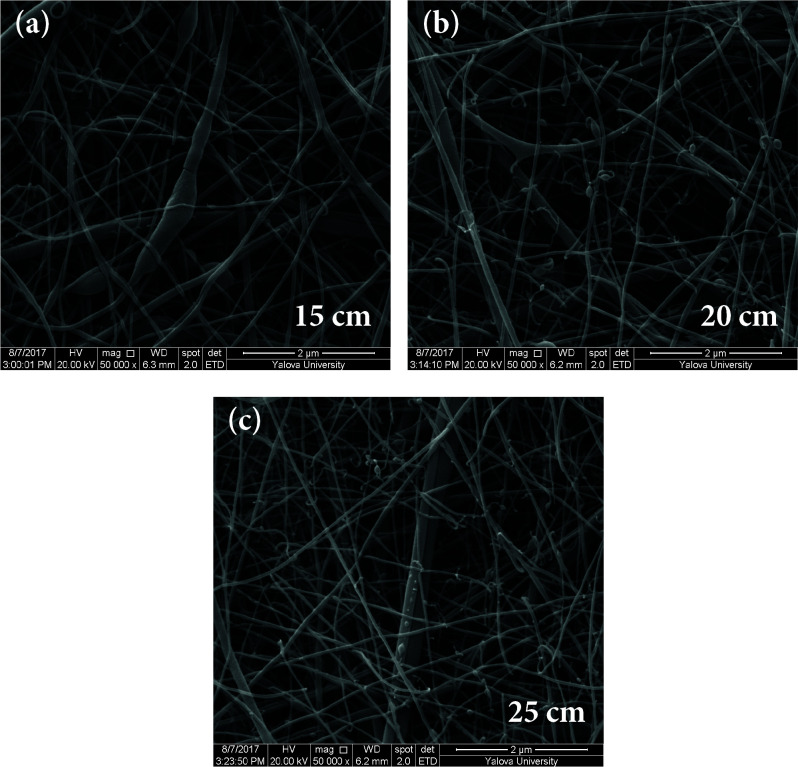
SEM images of silk fibroin nanofiber mats with 10% by weight and applied voltage 35 kV [15 cm (a), 20 cm (b) and 25 cm (c), respectively] ( 50000).

As shown in Figures 5–7, the thinnest measured diameters value of silk fibroin nanofibers was 33 ± 2 nm (40 kV, 25 cm), the thickest measured diameter value of silk fibroin nanofibers was 62 ± 6 nm (37 kV, 15 cm). These produced silk fibroin nanofibers were continuous fiber, but some mats had staple fiber. 

**Figure 8:**
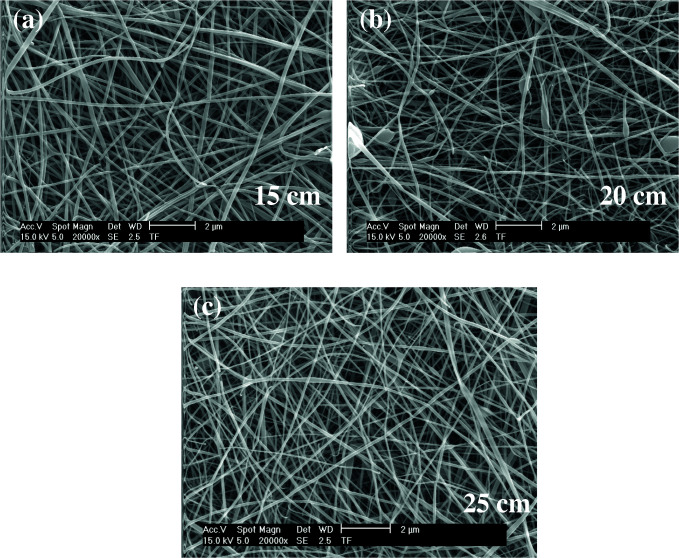
SEM images of silk fibroin nanofiber mats with 12.5% by weight and applied voltage 40 kV [15 cm (a), 20 cm (b) and 25 cm (c), respectively] ( 20000).

**Figure 9:**
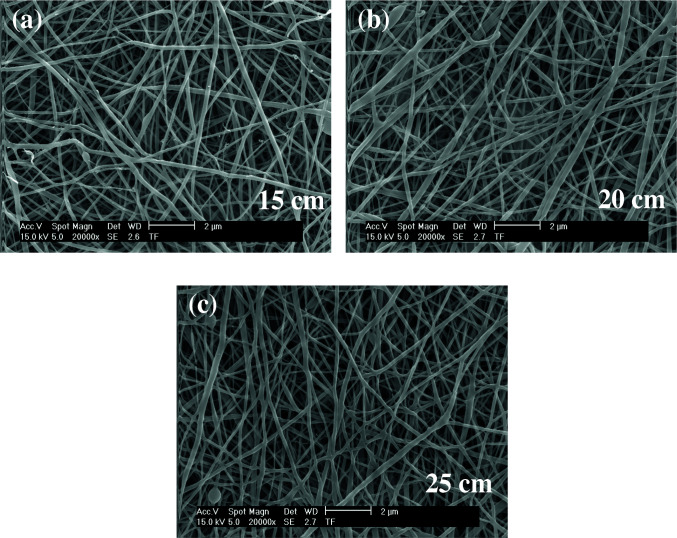
SEM images of silk fibroin nanofiber mats with 12.5% by weight and applied voltage 37 kV [15 cm (a), 20 cm (b) and 25 cm (c), respectively] ( 20000).

**Figure 10:**
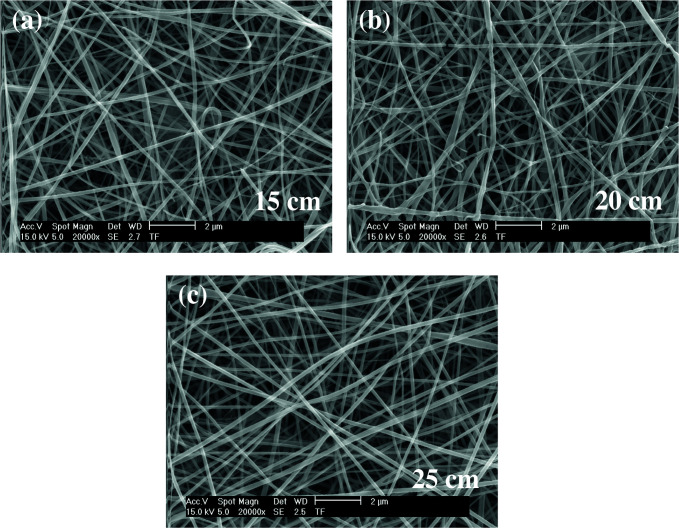
SEM images of silk fibroin nanofiber mats with 12.5% by weight and applied voltage 35 kV [15 cm (a), 20 cm (b) and 25 cm (c), respectively] ( 20000).

As shown in Figures 8–10, the thinnest measured diameter value of silk fibroin nanofibers was 55 ± 8 nm (40 kV, 25 cm), the thickest measured diameter value of silk fibroin nanofibers was 111 ± 14 nm (37 kV, 15 cm). These produced mats had intense fiber structure and continuous fiber. 

**Figure 11:**
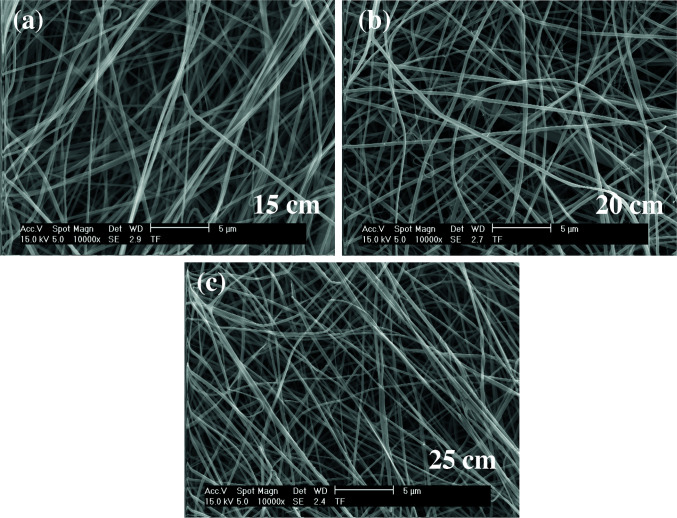
SEM images of silk fibroin nanofiber mats with 15% by weight and applied voltage 40 kV [15 cm (a), 20 cm (b) and 25 cm (c), respectively] ( 10000).

**Figure 12:**
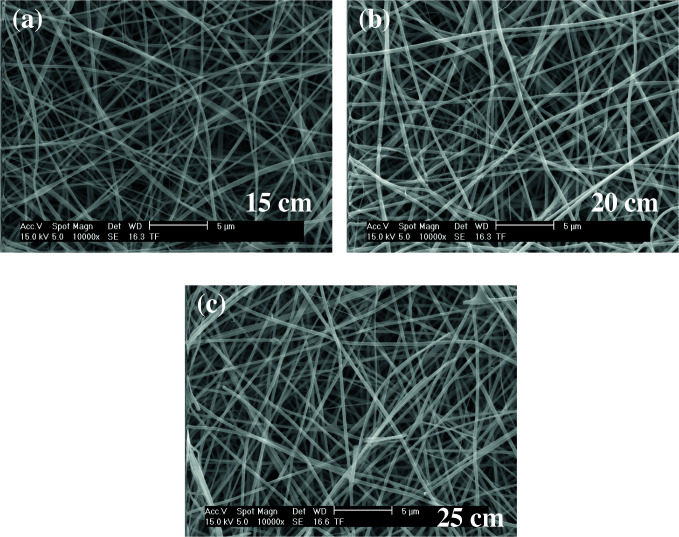
SEM images of silk fibroin nanofiber mats with 15% by weight and applied voltage 37 kV [15 cm (a), 20 cm (b) and 25 cm (c), respectively] ( 10000).

**Figure 13:**
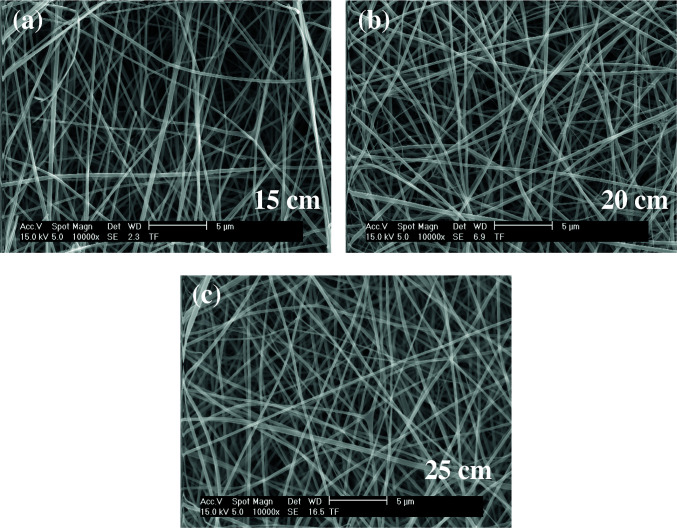
SEM images of silk fibroin nanofiber mats with 15% by weight and applied voltage 35 kV [15 cm (a), 20 cm (b) and 25 cm (c), respectively] ( 10000).

As shown in Figures 11–13, the thinnest measured diameter value of silk fibroin nanofibers was 147 ± 22 nm (40 kV, 25 cm), the thickest measured diameter value of silk fibroin nanofibers was 215 ± 26 nm (37 kV, 15 cm). These produced mats had intense than previous fiber structure. These nanofibers were continuous fiber. 

All produced silk fibroin nanofiber mats had the thinnest diameter value at 40 kV and 25 cm, and the thickest diameter value at 37 kV and 15 cm. High voltage and long distance are used for the thinnest diameter fiber. As mentioned before, due to the parameters that are effective in nanofiber mat production, a comparison with the values found in the literature was made considering the difference between these parameters. It was observed that they obtained nanofiber diameter in the range of 12–700–1500 nm at concentrations of 12–15–17% wt (2–4 kV, 12 cm) [25, 26]. 

### 3.5. The fiber diameter effect of applied voltage

**Figure 14:**
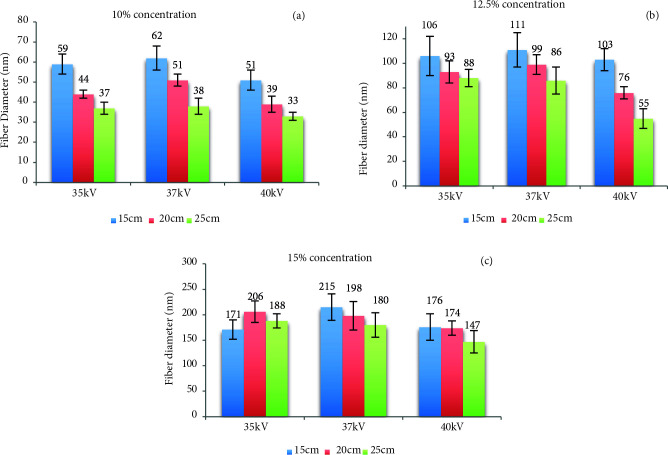
Fiber diameter value of silk fibroin nanofiber mats, 10% (a), 12.5% (b) and 15% (c) concentration.

The values given in Figure 14 were reached by grouping all the mats according to concentration differences. 

According to Figure 14, the thickest fiber diameters of silk fibroin nanofiber mats were occurred at 15% concentration, and the thinnest silk fibroin fiber diameters of were obtained at 10% concentration. Therefore, the concentration changes were affected in parallel with fiber diameter values. When the distances were investigated, fiber diameter values decreased in all concentration depending on the increase in distance. As mentioned in the evaluation of SEM images, although one-to-one comparison cannot be made due to different parameters, they obtained nanofiber diameters between 12 and 1500 nm at similar concentrations [25,26].

### 3.6. In vitro cell viability analysis

**Figure 15:**
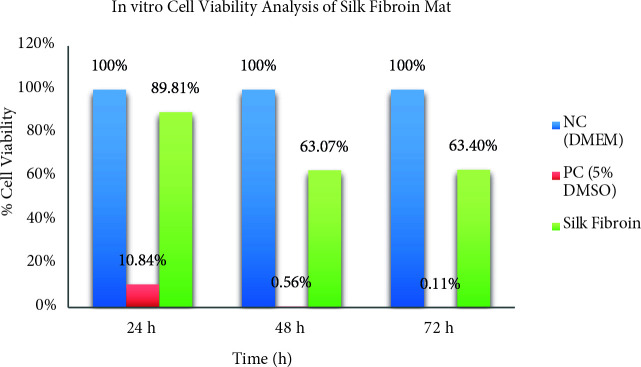
In vitro cell viability analysis of silk fibroin mat.

The extracts were incubated in 100% concentration culture media, which were 37 °C, 5% CO_2_. The samples were observed at 24, 48 and 72 h. The tested silk fibroin mat was indicated by comparison negative control as cell viability percentage. The cell viability percentage of silk fibroin mat was indicated in Figure 15. 

According to the results in Figure 15, at cell viability of silk fibroin mat was occurred high cell viability after 24 h, but it was obtained low cell viability at the 48th h. When the 48th h and 72th h were reached, there was a slight increase in cell viability. It was observed that silk fibroin provided cell viability up to 3–7 days and 3 weeks (silk fibroin with endothelial cells) according to different test hours [39–42]. As a result of the test, it has been observed that silk fibroin was suitable for cell proliferation after 72 h, and it has been identified with the literature.

## 4. Conclusion

In accordance with the test results obtained in this study, silk fibroin fiber diameters decreased with distance increase, and thinner fibers were produced. The reason for this is that the applied voltage to which the nanofibers are exposed until they reach the collector is continuous and the silk fibroin fibers are got over by thinning. This condition was observed similarly at three concentrations. Also, silk fibroin fiber diameter increased in direct proportion to depending on concentration rise. Therefore, the necessity has been determined low concentration for fine silk fibroin fiber production and high concentration for thick fiber production.

Thickness of produced silk fibroin mats was decrease depending on distance rise in 10% and 12.5% concentration production sets. The silk fibroin mat thickness was increased with solution concentration rise. It has been determined that close-range was affected by mat thickness. If the mat thickness was intended to be higher, optimal thickness was observed at a concentration of 12.5%, 37 kV applied voltage and 25 cm distance. The silk fibroin mat thickness increased with distance rise due to environmental parameters at 15% concentration production. 

When all the results were examined, it was found that 12.5% concentration was a suitable concentration in order to obtain the most strength silk fibroin nanofiber mats. The strength was affected inversely proportional the applied voltage rises and distance. The best result was obtained at 40 kV applied voltage and 15 cm distance. 

When SEM figures were analysed, at 10% concentration, the ratio of staple fiber increased with applied voltage decreasing. The most uniform silk fibroin fibers were obtained 37 kV applied voltage and 25 cm distance. At 12.5% concentration, silk fibroin fibers of mat were regular and a little beaded depending on applied voltage decreasing. The best silk fibroin fibers were obtained 35 kV applied voltage and 25 cm distance. At 15% concentration, it was found that there was more fiber density than the fiber density formed at other concentrations. In this concentration, silk fibroin fibers of all mats obtained were uninterrupted and uniform. It was determined that pilling and fiber breaks decreased with increasing solution concentration and viscosity. 

As a result of data obtained, it was found that the parameters of strength, mat thickness, surface of mats and fiber diameter were influenced by concentration, distance and applied voltage. When the test results of products made under the working conditions were examined, it was found that 10% concentration, 40 kV applied voltage and 25 cm distance were used to produce the finest fibers in terms of fiber fineness. From this point of view, if the production of fine fibers is aimed, high applied voltage and distance in addition to low concentration are very effective. If the thickness of mat is aimed to be more than, 12.5% concentration, 37 kV applied voltage and 15–20 cm distance are used to produce the most optimum thick mat. When the results were examined in terms of strength, it was determined that 12.5% concentration, 40 kV applied voltage and 15 cm distance were found for the most strength mat. At cell viability of silk fibroin mat was occurred high cell viability after 24 h, but it was obtained low cell viability at the 48th hours. When the 48th h and 72th h were reached, there was a slight increase in cell viability.
